# A cross-sectional analysis of polycyclic aromatic hydrocarbons and diesel particulate matter exposures and hypertension among individuals of Mexican origin

**DOI:** 10.1186/s12940-015-0039-2

**Published:** 2015-06-12

**Authors:** Komal S. Bangia, Elaine Symanski, Sara S. Strom, Melissa Bondy

**Affiliations:** 1 Office of Environmental Health Hazard Assessment, 1515 Clay St. Suite 1600, Oakland, CA 94612 USA; 2 Department of Epidemiology, Human Genetics and Environmental Sciences, Southwest Center for Occupational and Environmental Health, 1200 Herman Pressler St. Suite W-1028, Houston, TX 77030 USA; 3 Department of Epidemiology, The University of Texas MD Anderson Cancer Center, Unit 1340, 1155 Pressler Street, Houston, TX 77030-4009 USA; 4 Baylor College of Medicine, One Baylor Plaza, Houston, TX 77030 USA

**Keywords:** Cardiovascular disease, Diesel particulate matter, Hypertension, Polycyclic aromatic hydrocarbons

## Abstract

**Background:**

Epidemiological studies have found that particulate matter is associated with increases in blood pressure. Yet, less is known about the effects of specific sources or constituents of particulate matter, such as diesel particulate matter or polycyclic aromatic hydrocarbons (PAHs). We evaluated associations between self-reported hypertension and residential air levels of diesel particulate matter and PAHs among individuals of Mexican origin living in a large inner city.

**Methods:**

The Mano a Mano cohort (established in 2001 by the University of Texas MD Anderson Cancer Center) is comprised of individuals of Mexican origin residing in Houston, Texas. Using geographical information systems, we linked modeled annual estimates of PAHs and diesel particulate matter at the census tract level from the 2002 and 2005 U.S. Environmental Protection Agency’s National-Scale Air Toxics Assessment to baseline residential addresses of cohort members who enrolled from 2001 to 2003 or 2004 to 2006, respectively. For each enrollment period, we applied mixed-effects logistic regression models to determine associations between diesel particulate matter and PAHs, separately, and self-reported hypertension while adjusting for confounders and the clustering of observations within census tracts and households.

**Results:**

The study population consisted of 11218 participants of which 77 % were women. The mean participant age at baseline was 41 years. Following adjustment for age, there was a dose-dependent, positive association between PAHs and hypertension (medium exposure, adjusted odds ratio (OR) = 1.09, 95 % CI: 0.88-1.36; high exposure, OR = 1.40, 95 % CI: 1.01-1.94) for individuals enrolled during 2001–2003; associations were generally similar in magnitude, but less precise, following adjustment for age, gender, smoking, and BMI. No association was detected for the later period. There was no evidence of an association between residential levels of diesel particulate matter and hypertension.

**Conclusions:**

This study builds on a limited number of prior investigations of the association between ambient air levels of PAHs or diesel particulate matter and hypertension by focusing on a relatively young cohort of predominantly adult women of Mexican origin. Future analyses are warranted to explore associations in the cohort using incident hypertension when sufficient data become available and to further examine associations between specific chemical constituents of particulate matter and hypertension in this and other populations.

## Background

A major risk factor for cardiovascular disease (CVD), the leading cause of death among Hispanics [1] and worldwide [2], is hypertension. Research has shown that elevations of 10 mmHg diastolic blood pressure (BP) and 5 mmHg systolic BP are associated with substantial (as large as 50 %) increases in risks for CVD [3]. Hypertension is known as a “silent killer” because most people do not have symptoms, yet consequences can be deadly as Hispanics experience difficulty in managing hypertension that may be due, in part, to access to medical care or not having health insurance [4, 5].

Ambient particulate matter (PM) ranks highly among causes of death worldwide (13^th^), taking the lives of about 800,000 individuals per year [3]. An elevation of 10 μg/m^3^ of fine particulate matter (particulate matter 2.5 microns or less in aerodynamic diameter) can increase BP by up to 5 mmHg [6], increasing risk for CVD, over time, by up to 76 % [3]. Specifically, PM can increase an individual’s BP by initiating inflammation and oxidative stress in the lungs and then causing vasoconstriction and endothelial damage. However, epidemiological studies have been equivocal. Some have demonstrated that PM exposure increases BP [3, 6-20], while a few researchers have shown an inverse or no association [3, 21-25]. This inconsistency suggests the need for research on specific sources of PM such as diesel particulate matter (DPM) or specific components of PM such as polycyclic aromatic hydrocarbons (PAHs).

Diesel particulate matter is a likely carcinogen [26] that is formed from combustion of diesel fuel. Similar to PM, numerous mechanisms have been suggested to explain how DPM exposure leads to elevated BP, such as systemic and pulmonary inflammation, an increase of pro-inflammatory cytokines, and oxidative stress that can impair endothelial function [27-32]. PAHs are a group of organic compounds (7 are known carcinogens) [33] and a component of PM formed as byproducts of incomplete combustion from sources such as fossil fuels and biomass. They can significantly contribute to increases in BP [34]. The biological mechanisms through which PAHs exert their effects on BP are similar to DPM, such as oxidative stress, local inflammatory effects, endothelial damage, and plaque promotion [35-43].

This cross-sectional investigation aimed to evaluate the association between exposures to residential air levels of PAHs and DPM and hypertension among a large cohort of adults of Mexican-origin who reside in Houston, Texas.

## Methods

### Study population

We used data collected from the Mexican American Cohort Study (MACS), also known as the “Mano a Mano” cohort study that was established by the Department of Epidemiology at the University of Texas MD Anderson Cancer Center (UTMDACC) in 2001. The Mano a Mano cohort is comprised of individuals of Mexican origin who at enrollment resided in Houston, TX for at least 1 year. Recruitment methods included random-digit dialing and block walking. All adult participants signed an informed consent and completed a baseline interview to seek information about their demographics, medical history, degree of acculturation, lifestyle habits, and work history. In this study, we restricted our analyses to a subset of the entire cohort, comprised of adults aged 18 years and older who enrolled during the period of 2001–2006 (*N* = 11218). Individuals from the same household were eligible to participate. Most households (5147) had one member, 2645 households had two members, 184 households had three members, 56 households had four members, and one household had five members. Our study was approved by The University of Texas Health Science Center, Committee for Protection of Human Subjects and by the MD Anderson Cancer Center Institutional Review Board.

### Outcome assessment

All participants were interviewed in person to determine their medical history including hypertension status. In the current study, we considered that a participant was hypertensive if they answered yes to the question: “have you been told by a health professional that you have high blood pressure or hypertension?”.

### Exposure assessment

Modeled estimates of ambient air levels of PAHs and DPM were extracted from the 2002 and 2005 National-Scale Air Toxics Assessment (NATA) developed by the U.S. Environmental Protection Agency (EPA) [44]. NATA provides information on more than 180 toxic air pollutants (181 in 2002 and 187 in 2005) based primarily on emissions from point sources, non-point sources, mobile sources, background sources, and sources from secondary formation (i.e., the transformation of one pollutant to another pollutant) in specific geographic areas [45, 46]. To generate the nationwide estimates of ambient air levels of toxic air pollutants (reported in units of μg/m^3^), depending on the source of emissions, NATA uses the National Emissions Inventory or the National Mobile Inventory Model (NMIM) to input data into two models, the Assessment System for Population Exposure Nationwide (ASPEN) and the Human Exposure Model 3 (HEM-3), respectively. In NATA, 16 PAHs are included among the broad classification of compounds termed Polycyclic Organic Matter (PAHPOM) [44].

Table 1 shows a breakdown of DPM and PAH modeled concentrations in percentiles. For analyses, we converted μg/m^3^ of PAHs to ng/m^3^. Using geographic information systems (Arc GIS, version 10 (ESRI, Redlands, CA)), the geocoded residences of participants who enrolled during 2001 to 2003 or 2004 to 2006 were linked to the 2002 or 2005 data, respectively.

**Table 1:** Residential air levels for diesel particulate matter (DPM) (μg/m^3^) and polycyclic aromatic hydrocarbons (PAHs) (ng/m^3^)

Enrollment period	Percentiles				
	5	25	50	75	95
DPM					
2001–2003	2.44	3.58	4.82	10.66	25.32
2004–2006	1.18	1.71	2.53	3.80	7.28
PAHs					
2001–2003	1.79	2.16	2.48	3.77	7.20
2004–2006	1.26	1.42	1.63	1.92	2.62

### Potential confounders

Information that was collected during the baseline interview included: date of birth, gender, comorbid conditions (previous diagnosis of diabetes, heart attack/disease, or high cholesterol), smoking status (current, former, and never), alcohol use (current, former, and never), body mass index (BMI) based on self-reported height and weight, educational attainment (bachelor degree and higher, high school graduate or GED, some high school, and no formal schooling or elementary or middle school), nativity status (U.S. born or Mexican born), length of residence at baseline address (<1 year, 1 to 5 years, > 5 years), number of years living in the U.S., physical activity (“active” if a participant engaged in strenuous exercise ≥ 1.5 h/week or moderate exercise ≥ 3 h/week; otherwise, “not active”), current employment status (currently working or currently not working), and degree of acculturation based on the Bidimensional Acculturation Scale for Hispanics (BAS) (high, BAS > 2.50 and low, BAS ≤ 2.50) [47].

### Statistical analysis

In preliminary analyses, we generated frequency distributions of socio-demographic and lifestyle factors among our study population. We performed chi-squared analyses to evaluate differences in categorical risk factors between enrollment periods, as well as simple logistic regression analyses to evaluate crude associations between covariates and hypertension status at a significance level of 0.05. We used multilevel mixed-effects logistic regression models to examine associations between PAHs or DPM and hypertension status. Air pollutant concentrations were modeled as both continuous and categorical variables, the latter based on the following categorization scheme: <50^th^, 50-89^th^, ≥90^th^ percentiles. We included random effects for census tract and household to account for correlated observations as individuals were nested within households and households were nested within census tracts. To assess for confounding, we applied multiple logistic regression models to determine associations between DPM and PAHs and hypertension while adjusting for the covariates listed above (see section on “Potential Confounders”), one at a time. Covariates were retained in the final model if their inclusion resulted in ≥ 10 % change in the estimate of the odds ratio [48].

Additionally, we compared associations for participants who lived at their baseline address for less than or equal to five years and for those that have lived at their baseline address for greater than five years because we expected less misclassification of exposure among individuals who lived at their residence for the longer time period. We also conducted a sensitivity analysis based on employment status at baseline because our exposure assessment that relied on residential location may have resulted in a different error structure for individuals who did not work outside of the home as compared to those who did. All statistical analyses were conducted using Intercooled STATA, version 11 (StataCorp LP, College Station, TX).

## Results

The study population consisted of 11218 participants from 8033 households enrolled between 2001 and 2006. In total, 13.1 % and 16.7 % of the cohort who enrolled in 2001–2003 or 2004–2006, respectively, reported having hypertension. Across both time periods, approximately 77 % of study participants were women and more than 50 % of participants were between 25 and 44 years of age. The mean age at baseline was 41 years. Most participants did not possess a high school degree (about 60 %) and approximately 60 % were currently employed. Table 2 provides a breakdown of the distribution of covariates for participants by hypertension status for each enrollment period. Except for age and degree of acculturation (as assessed by the BAS score), there were differences (*p* < 0.05) for all other selected covariates. Notably, the proportion of women who enrolled in the cohort was greater in 2004–2006 (82 %) as compared to 2001–2003 (72 %) and 73 % of cohort members were currently employed in the earlier time period while only 48 % were currently employed in the later time period.

**Table 2:** Distribution of characteristics for participants and crude associations with hypertension status by enrollment period (N = 11218)

Characteristic	2001–2003				2004–2006			
	Hypertension		OR	95 CI	Hypertension		OR	95 CI
	No	Yes			No	Yes		
Gender^**^								
Men	1237	156	Ref	Ref	943	207	Ref	Ref
Women	3053	489	1.32^*^	1.07 - 1.62	4294	839	0.9	0.74 - 1.09
Age								
18–34	2021	31	Ref	Ref	2445	97	Ref	Ref
35–54	1680	240	9.60^*^	6.52 - 14.14	2142	419	5.42^*^	4.20 - 6.99
55–74	589	374	45.39^*^	29.78 - 69.17	650	530	26.37^*^	18.87 - 36.86
Level of education^**^								
Middle School or less	1523	371	2.78^*^	2.12 - 3.64	1780	574	2.77^*^	2.13 - 3.60
Some High School/High School Graduate/GED	1743	172	1.00	0.76 - 1.33	2571	349	0.97	0.75 - 1.25
More than High School	1024	102	Ref	Ref	882	122	Ref	Ref
Missing	0	0	NA	NA	4	1	NA	NA
Currently employed^**^								
No	233	24	0.64	0.41 - 1.00	2282	496	1.20^*^	1.02 - 1.42
Yes	3097	493	Ref	Ref	2563	475	Ref	Ref
Missing	960	128	NA	NA	392	75	NA	NA
Years living at baseline address^**^								
<1	668	54	Ref	Ref	1009	108	Ref	Ref
1–5	1718	146	1.07	0.75 - 1.52	2249	326	1.39^*^	1.07 - 1.81
>5	1902	445	3.23^*^	2.32 - 4.49	1979	612	3.32^*^	2.56 - 4.31
Missing	2	0	NA	NA	0	0	NA	NA
High cholesterol diagnosis^**^								
Yes	273	171	6.41^*^	4.86 -8.43	528	364	6.88^*^	5.35 - 8.86
No	4017	474	Ref	Ref	4709	682	Ref	Ref
Heart attack diagnosis^**^								
Yes	15	12	5.92^*^	2.41 - 14.53	19	36	14.72^*^	7.15 - 30.31
No	4275	633	Ref	Ref	5218	1010	Ref	Ref
Diabetes diagnosis^**^								
Yes	365	241	6.89^*^	5.49 - 8.66	470	385	7.74^*^	6.09 - 9.84
No	3925	404	Ref	Ref	4767	661	Ref	Ref
Smoking status^**^								
Current	630	75	0.82	0.62 - 1.09	637	115	0.94	0.73 - 1.21
Former	565	155	2.03^*^	1.61 - 2.57	667	194	1.57^*^	1.27 - 1.94
Never	2916	415	Ref	Ref	3897	737	Ref	Ref
Missing	179	0	NA	NA	36	0	NA	NA
Alcohol use^**^								
Current	1157	113	0.58	0.46 - 0.74	1204	182	0.71	0.57 - 0.87
Former	431	129	1.97^*^	1.53 - 2.54	430	164	2.16^*^	1.68 - 2.77
Never	2524	403	Ref	Ref	3487	698	Ref	Ref
Missing	178	0	NA	NA	116	2	NA	NA
Physical activity level^**^								
Active	1160	110	Ref	Ref	1209	176	Ref	Ref
Not active	3099	530	1.91^*^	1.50 - 2.41	4002	864	1.58^*^	1.29 - 1.94
Missing	31	5	NA	NA	26	6	NA	NA
BMI (kg/m^2^)^**^								
Underweight/Normal (<24.9)	1020	82	Ref	Ref	1180	102	Ref	Ref
Overweight (25.0–29.9)	1559	187	1.55^*^	1.16 - 2.09	1798	331	2.40^*^	1.83 - 3.16
Obese I-III (≥30.0)	1674	375	3.13^*^	2.36 - 4.15	2115	588	3.96^*^	3.02 - 5.20
Missing	37	1	NA	NA	144	25	NA	NA
Degree of acculturation								
High level of acculturation (BAS > 2.50)	1583	219	0.83	0.68 - 1.01	1903	377	0.95	0.80 - 1.12
Low level of acculturation (BAS ≤ 2.50)	2699	426	Ref	Ref	3317	665	Ref	Ref
Missing	8	0	NA	NA	17	4	NA	NA
No. of years living in the U.S.^**^								
≤9	1207	63	Ref	Ref	1613	158	Ref	Ref
10–14	614	42	1.34	0.88 - 2.03	845	96	1.18	0.88 - 1.59
≥15	2469	540	4.54^*^	3.39 - 6.08	2778	792	3.34^*^	2.68 - 4.15
Missing	0	0	NA	NA	1	0	NA	NA
Nativity^**^								
U.S. born	1288	255	1.52^*^	1.26 - 1.84	1347	368	1.66^*^	1.39 - 1.97
Mexico born	3001	390	Ref	Ref	3874	676	Ref	Ref
Other	1	0	NA	NA	16	2	NA	NA

*Abbreviations*: *CI* confidence interval, *OR* odds ratio, *NA* not applicable

^*^p < 0.05 for associations between covariates and hypertension status (crude odds ratio)

^**^p < 0.05 for associations between covariates and enrollment period (chi-squared analysis)

In simple logistic regression analyses (see Table 2), odds of prevalent hypertension increased with age, years living at the baseline address, and BMI. Increased odds of hypertension were also detected among persons with diagnoses of high cholesterol, a prior heart attack, or diabetes, as well as those who were physically inactive. We detected higher odds for hypertension among U.S. born individuals as compared to those born in Mexico; odds also increased with the number of years living in the U.S. Women experienced higher odds of hypertension as compared to men enrolled in 2001-2003, but not in 2004-2006.

Age at baseline was the only variable identified as a confounder for the association between DPM and hypertension. Gender, smoking, and BMI, risk factors of hypertension were then added to the model to determine if joint confounding was evident. Results indicated less than a 10 % difference in the odds ratios when comparing the results for the age-adjusted model to the fully adjusted model. Nonetheless, for completeness, we present the fully-adjusted results along with prevalence odds ratios (ORs) and 95 % Confidence Intervals (CIs) for the association between hypertension and DPM adjusted only for the random effects (census tracts and households) and age at baseline in Table 3.

**Table 3:** Odds Ratios (95 % CIs) for the associations between hypertension and diesel particulate matter (DPM) (μg/m^3^)

Enrollment period	DPM^a^	N	OR ^b^	95 % CI	OR^c^	95 % CI	OR^d^	95 % CI
2001–2003	Referent (1.39 – 4.81)	2450	---	---	---	---	---	---
	Medium (4.82 – 17.69)	1987498	1.31^*^	1.07 – 1.61	1.15	0.92 – 1.42	1.13	0.91 – 1.41
	High (17.70 – 25.32)		1.13	0.81 – 1.58	1.10	0.77 – 1.57	1.01	0.71 – 1.45
	Continuous (per μg/m^3^)		1.01	0.99 – 1.03	1.01	0.99 – 1.02	1.00	0.99 – 1.02
2004–2006	Referent (0.75 – 2.52)	2874	---	---	---	---	---	---
	Medium (2.53 – 5.82)	2735	1.29^*^	1.04 – 1.61	1.03	0.85 – 1.25	1.05	0.86 – 1.29
	High (5.83 – 8.77)	674	1.14	0.80 – 1.62	0.92	0.67 – 1.27	0.84	0.61 – 1.17
	Continuous (per μg/m^3^)		1.03	0.98 – 1.10	0.99	0.94 – 1.04	0.98	0.93 – 1.03

*Abbreviations*: *CI* confidence interval, *OR* odds ratio

^*^Significant at *p* < 0.05

^a^Referent: < 50^th^ percentile, Medium: 50-89^th^ percentile, High: ≥ 90^th^ percentile

^b^Adjusted for census tract and household as random effects

^c^Adjusted for age at baseline and census tract and household as random effects

^d^Adjusted for age at baseline, gender, smoking, and BMI and census tract and household as random effects

For the 2001-2003 enrollment period with only random effects included in the model, the odds of hypertension were elevated for DPM in the medium and high exposure groups when compared to the referent category (OR = 1.31, 95 % CI: 1.07-1.61; OR = 1.13, 95 % CI: 0.81-1.58). Following additional adjustment for age at baseline, odds ratios (medium exposure category: OR = 1.15, 95 % CI: 0.92-1.42; high exposure category: OR = 1.10, 95 % CI: 0.77-1.57) diminished somewhat in magnitude and results were less precise. For the 2004-2006 enrollment period, we observed odds ratios of 1.29 (95 % CI: 1.04-1.61) and 1.14 (95 % CI = 0.80-1.62) for the medium and high exposure groups, respectively. Following adjustment for age, the ORs were close to the null (medium: OR = 1.03; 95 % CI = 0.85-1.25; high: OR = 0.92; 95 % CI = 0.67 - 1.27). For both enrollment periods, associations were generally similar in magnitude, but less precise, following adjustment for age, gender, smoking, and BMI. No associations in any of the models were detected when DPM was modeled as a continuous variable.

Similar to DPM, age at baseline was the only variable identified as a confounder for PAHs. Gender, smoking, and BMI were also added to this model, but similarly to DPM, there was little evidence of joint confounding. Results for PAHs are reported in Table 4. Following adjustment for the random effects for census tracts and households, we observed elevated odds for hypertension in the medium (OR = 1.09; 95 % CI: 0.89-1.34) and high (OR = 1.56; 95 % CI = 1.16-2.11) exposure groups for those members enrolled in the earlier (2001–2003) enrollment period (Table 4). Following additional adjustment for age at baseline, odds ratios remained elevated for both exposure groups (OR = 1.09; 95 % CI = 0.88-1.36 for the medium exposure group and OR = 1.40; 95 % CI: 1.01-1.94 for the high exposure group). After adjusting for age, gender, smoking, and BMI, associations were similar in magnitude, albeit less precise. Additionally, for the 2001-2003 enrollment period in the age-adjusted model, the odds for hypertension also increased by 5 % (OR = 1.05, 95 % CI: 1.00-1.12) for a one unit increase (ng/m^3^) in ambient PAH levels. For the 2004-2006 enrollment group, no associations between PAHs and hypertension were detected.

**Table 4:** Odds Ratios (95 % CIs) for the associations between hypertension and polycyclic aromatic hydrocarbons (PAHs) (ng/m^3^)

Enrollment period	PAHs^a^	N	OR^b^	95 % CI	OR^c^	95 % CI	OR^d^	95 % CI
2001–2003	Referent (0.92 – 2.47)	2338	---	---	---	---	---	---
	Medium (2.48 – 4.76)	2083	1.09	0.89 – 1.34	1.09	0.88 – 1.36	1.07	0.85 – 1.33
	High (4.77 – 12.38)	514	1.56^*^	1.16 – 2.11	1.40^*^	1.01 – 1.94	1.32	0.95 – 1.83
	Continuous (per ng/m^3^)		1.07^*^	1.02 – 1.13	1.05^*^	1.00 – 1.12	1.04	0.98 – 1.10
2004–2006	Referent (0.91 – 1.62)	3112	---	---	---	---	---	---
	Medium (1.63 – 2.30)	2511	0.96	0.78 – 1.19	0.90	0.74 – 1.09	0.87	0.72 – 1.06
	High (2.31 – 12.76)	660	0.79	0.56 – 1.11	0.96	0.71 – 1.31	0.97	0.71 – 1.33
	Continuous (per ng/m^3^)		1.01	0.91 – 1.12	0.99	0.90 – 1.08	0.99	0.90 – 1.09

*Abbreviations*: *CI* confidence interval, *OR* odds ratio

^*^Significant at *p* < 0.05

^a^Referent: < 50^th^ percentile, Medium: 50-89^th^ percentile, High: ≥ 90^th^ percentile

^b^Adjusted for census tract and household as random effects

^c^Adjusted for age at baseline and census tract and household as random effects

^d^Adjusted for age at baseline, gender, smoking, and BMI and census tract and household as random effects

For the 2001-2003 enrollment group living more than 5 years at their baseline address, age-adjusted odds ratios were elevated for the medium (OR = 1.12, 95 % CI: 0.86 - 1.47) and high (OR = 1.33, 95 % CI: 0.90 - 1.97) exposure groups. For persons living five years or less at their baseline address, age-adjusted odds ratios were 1.05 (95 % CI: 0.72 - 1.54) and 1.68 (95 % CI: 0.93 - 1.13) for the medium and high exposed groups, respectively. For persons who did not work outside of the home, odds ratios were elevated but extremely imprecise due to small sample sizes among those with hypertension (*n* = 24) and hence the results are not shown.

## Discussion

While epidemiological studies have found that particulate matter is associated with increases in blood pressure, less is known about the effects of specific sources or constituents of PM, such as diesel particulate matter or polycyclic aromatic hydrocarbons. In our cross-sectional evaluation of the associations between hypertension and residential air levels of DPM and PAHs among individuals of Mexican-origin, our results indicate an association between PAHs and hypertension for individuals enrolled in the 2001-2003 time period, but not in the later time period. There were little to modest differences in the associations when the cohort was stratified on the basis of how long participants lived at their current addresses at baseline. No associations were detected for DPM in either time period.

Our positive findings for an association between PAHs and hypertension agree with the panel study by Jacobs et al. [49], who concluded that chrysene-5,6-dione and benzo[a]pyrene-3,6-dione can increase systolic BP and pulse pressure. In addition, animal studies have demonstrated that arterial BP can increase due to exposure to a PAH-contained organic mixture derived from coal [50], as well as to specific PAHs, benzo[a]pyrene (BaP), and dimethylbenz[a]anthracene (DMBA) [36, 51, 52]. Another study, however, showed a lack of association between PAHs and BP in rats [53], but was criticized for its BaP administration and BP monitoring methods [36]. Contrary to the positive associations reported in Cosselman et al. [54] and Nemmar et al. [30], our results did not provide evidence for an association between DPM and hypertension for individuals in either enrollment period. However, our findings are similar to the four previous randomized, double-blind, cross-over studies conducted on DPM and cardiopulmonary effects that concluded that DPM had no significant effect on BP [28, 29, 55, 56].

We observed a dose-dependent association between PAHs and hypertension among individuals enrolled in the 2001-2003 time period, but no association for individuals who enrolled in the 2004-2006 period. Differences in characteristics of the study population between enrollment periods may explain the equivocal findings. There were more women enrolled in the cohort in 2004-2006 (who appear to have a more modest association between PAHs and hypertension than men - see discussion below) as compared to 2001-2003, and a significantly greater proportion of participants were currently employed in the earlier time period than in the later time period. Secondly, there were differences in the distributions of PAH modeled concentrations by time period (the interquartile range for PAHs in 2002 was 1.61 ng/m^3^ as compared to 0.5 ng/m^3^ in 2005) that may also explain the equivocal findings. While such differences may be due to the change in methodology that NATA used to generate the two data releases, a decline in emissions or both [45, 46], decreased levels of variability in the 2005 NATA data likely diminished the comparisons that could be made between exposure groups.

Based on the results from our sensitivity analyses between those who lived at their residential address for five or more years as compared to those who lived at their address for less than 5 years, there appeared to be a modest increase in the odds ratio for the longer-term residents when comparing the high exposure group (but not the middle exposure group) to the low exposure group. Results from previous studies that evaluated associations between PM exposures and CVD outcomes produced equivocal results, with some studies reporting little differences based on length of residence whereas others reported stronger associations for participants who lived for longer periods at their current residential addresses [57, 58]. Associations between roadway traffic noise and hypertension were also stronger for residents living at their current addresses for more than 10 years [59].

In post-hoc analyses, we further explored the potential for effect measure modification due to sex, smoking, and age for the association between PAHs and hypertension among individuals enrolled in the earlier time period (Table 5). While we recognize that our study was underpowered to evaluate effect modification, interesting results emerged. Both men and women showed dose response relationships between PAH exposure and hypertension status. Differences were also detected on the basis of smoking status, with the strongest association between hypertension and high level of PAH exposure observed among former smokers. When evaluating potential differences by age at enrollment, stronger associations were observed for individuals in the youngest (18–34 years) age category.

**Table 5:** Associations between PAHs (ng/m^3^) and hypertension by age, gender, and smoking status (2002 enrollment period)

Potential covariate	PAHs^a^	OR	95 % CI
Age^b^			
18-34 (*n* = 2052)	Referent (0.92 – 2.47)	---	
	Medium (2.48 – 4.76)	1.35	0.63 - 2.89
	High (4.77– 12.38)	1.70	0.54 - 5.31
	Continuous (per ng/m^3^)	1.05	0.87 - 1.27
35-54 (*n* = 1920)	Referent (0.92 – 2.47)	---	
	Medium (2.48 – 4.76)	1.01	0.74 - 1.40
	High (4.77 – 12.38)	1.45	0.91 - 2.33
	Continuous (per ng/m^3^)	1.06	0.98 - 1.15
55-74 (*n* = 963)	Referent (0.92 – 2.47)	---	
	Medium (2.48 – 4.76)	1.09	0.81 - 1.47
	High (4.77 – 12.38)	1.30	0.84 - 2.02
	Continuous (per ng/m^3^)	1.05	0.97 - 1.14
Gender^c^			
Men (*n* = 1393)	Referent (0.92 – 2.47)	---	---
	Medium (2.48 – 4.76)	1.51	0.67 – 3.39
	High (4.77 – 12.38)	2.03	0.52 – 7.97
	Continuous (per ng/m^3^)	1.09	0.87 – 1.37
Women (*n* = 3542)	Referent (0.92 – 2.47)	---	---
	Medium (2.48 – 4.76)	1.06	0.80 - 1.42
	High (4.77 – 12.38)	1.41	0.93 – 2.15
	Continuous (per ng/m3)	1.06	0.99 – 1.14
Smoking^c^			
Never (*n* = 3331)	Referent (0.92 – 2.47)	---	---
	Medium (2.48 – 4.76)	1.00	0.76 – 1.32
	High (4.77 – 12.38)	1.02	0.67 – 1.56
	Continuous (per ng/m^3^)	1.01	0.94 – 1.09
Current (*n* = 705)	Referent (0.92 – 2.47)	---	---
	Medium (2.48 – 4.76)	1.30	0.75 – 2.25
	High (4.77 – 12.38)	1.84	0.81 – 4.18
	Continuous (per ng/m^3^)	1.10	0.95 – 1.27
Former (*n* = 720)	Referent (0.92 – 2.47)	---	---
	Medium (2.48 – 4.76)	1.41	0.69 – 2.86
	High (4.77 – 12.38)	4.99^*^	1.35 – 18.46
	Continuous (per ng/m^3^)	1.18	0.97 – 1.44

*Abbreviations*: *CI* confidence interval, *OR* odds ratio

^*^Significant at *p* < 0.05

^a^Referent: < 50th percentile, Medium: 50-89th percentile, High: ≥ 90th percentile

^b^Adjusted for census tract and household as random effects

^c^Adjusted for age at baseline and census tract and household as random effects

An advantage of the current study was that it built upon a large cohort of relatively young individuals, mostly women, of Mexican origin who live in Houston, TX. Also, as result of having data on cohort members collected by interview, this study provided a substantial amount of information on potential factors that may operate as confounders. Houston is the fourth largest metropolitan city in the U.S., is one of the busiest seaports, has considerable amounts of traffic, and is home to the largest petrochemical complex in the country. Houstonians are exposed to more air pollutants than individuals residing in most other U.S. cities [60], and therefore, the city of Houston was an ideal location for this study. While inferences are limited to individuals of Mexican origin, research has shown that Hispanics have poorer cardiovascular profiles [60], lower socioeconomic status, increased all-cause mortality rates, and are less likely to have health insurance than other ethnic groups [5]. These individuals represent a rapidly growing population (60 % of the Hispanic population), which has received little attention regarding health effects due to air pollutant exposure in Houston, TX.

Limitations of this study include analyzing prevalent rather than incident disease, which calls into question the temporal relationship between the two primary exposures of interest (PAHs and DPM) and hypertension. As previously noted, the Mano a Mano cohort is predominantly female and young and hence our results may not be generalizable to males or to an older population. For our outcome assessment, cohort members self-reported their hypertension status. Research has been inconsistent in determining whether self-reporting hypertension is an accurate measure of hypertension [61-63]. We would have preferred to use measured blood pressure, prescription history for anti-hypertensive medications, or physicians’ reports to determine hypertension status, but such data were not available. We cannot rule out information bias because participants who live in neighborhoods with poorer air quality may have been more likely to self-report having hypertension due to living in worse built environments and exposures to myriad psychosocial stressors. Finally, it is possible that some younger women were reporting gestational hypertension in response to the question about having hypertension, but the questionnaire did not differentiate pregnancy-induced hypertension from other types.

Generating exposure estimates from modeled rather than monitored assessments has strengths and limitations. Using data from NATA allowed us to account for a variety of stationary and mobile sources along with providing the ability to have specific components of PM measured [45, 46]. However, NATA does not model indoor exposure sources or account for individual behaviors and activities, which may contribute significantly to personal exposures. Studies on comparisons between indoor, outdoor, and personal exposures have found mixed results [64-66]. While questions remain whether NATA modeled estimates can appropriately act as surrogates for personal exposures to PAHs and DPM, there are limited epidemiologic investigations assessing the influence of outdoor air levels of PAHs and DPM on hypertension and the use of this secondary data was a cost-effective first approach in assessing these associations.

## Conclusions

To our knowledge, this is among the first studies to analyze the relationship between ambient levels of two air pollutants, PAHs and DPM, and self-reported hypertension among relatively young individuals, mostly women, of Mexican origin. Our multiple logistic regression analysis suggests that ambient levels of PAHs were associated with hypertension for the 2001-2003 enrolled individuals and did show a dose response relationship. No associations between ambient levels of DPM and odds of hypertension were observed. Future analyses are warranted to further explore associations in the Mano a Mano cohort using incident hypertension when sufficient data become available and to explore risks of hypertension with specific chemical constituents (e.g., metals and organic carbon) of particulate matter.

## Abbreviations

BAS: Bidimensional Acculturation Scale for Hispanics
BMI: Body mass index
BP: Blood pressure
CVD: Cardiovascular disease
DPM: Diesel particulate matter
NATA: National Air Toxics Assessment
PAHs: Polycyclic aromatic hydrocarbons
PM: Particulate matter
